# Trophic and Environmental Generalists Maintain Pollinator Network Functional Continuity by Switching Modules Through Time

**DOI:** 10.1002/ece3.74263

**Published:** 2026-09-17

**Authors:** Hai‐Dong Li, Yan‐Hui Zhao, Marcel Holyoak, Zhibin Tao, Kun Xu, Zhikun Wu, Hong Wang, De‐Zhu Li

**Affiliations:** ^1^ Key Laboratory for Plant Diversity and Biogeography of East Asia Kunming Institute of Botany, Chinese Academy of Sciences Kunming China; ^2^ Department of Environmental Science and Policy University of California Davis California USA; ^3^ Key Laboratory of Aquatic Botany and Watershed Ecology, Wuhan Botanical Garden Chinese Academy of Sciences Wuhan China; ^4^ Lijiang Forest Biodiversity National Observation and Research Station Kunming Institute of Botany, Chinese Academy of Sciences Lijiang China; ^5^ Department of Pharmacy Guizhou University of Traditional Chinese Medicine Guiyang China; ^6^ Plant Germplasm and Genomics Center, Germplasm Bank of Wild Species Kunming Institute of Botany, Chinese Academy of Sciences Kunming China; ^7^ Center for Interdisciplinary Biodiversity Research and College of Forestry Shandong Agricultural University Tai'an China

**Keywords:** multilayer networks, niche spaces, plant‐pollinator networks, seasonal dynamics, species roles, temporal persistence

## Abstract

Plant‐pollinator networks are temporally dynamic and crucial for ecosystem functioning. Yet, the characteristics of species that allow networks to persist through a season remain poorly understood. We investigated plant‐pollinator interactions through an entire flowering season at four alpine meadows in SW China with a temporal multilayer network approach, where subnetworks representing successive time periods (layers) are interconnected via interlayer links. We show that temporal multilayer networks were highly modular, and that pollinator species' importance (versatility) in persistence through time of the multilayer network was correlated with their trophic and environmental niche space. Highly versatile pollinators persisted through time by switching between and occupying up to six network modules through time. These species with high versatility also had broad environmental and trophic niches, allowing them to maintain network functional stability throughout the flowering season. Future studies should investigate whether changes in plant–pollinator associations arise from active foraging decisions, such as switching among plant species to maximize resource acquisition or maintaining fidelity to preferred partners, or instead from environmentally and phenologically driven changes in plant availability and composition through seasons.

## Graphical abstract

By linking niche breadth to temporal pollination networks, we move beyond documenting dynamics to reveal the functional processes that maintain network persistence. Our findings directly identify the versatile generalist species that are vital for stability, offering clear priorities for conservation in a changing climate.

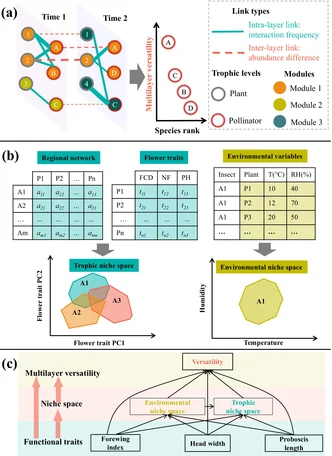

## 1 Introduction

Interactions between plant and animal species form ecological networks that are crucial in maintaining biodiversity and ecosystem functioning (Bascompte and Jordano 2007; Bastolla et al. 2009; Pocock et al. 2012). These networks are inherently dynamic, with species and interactions changing over time (Baldock et al. 2011; Burkle et al. 2013; CaraDonna et al. 2021; Olesen et al. 2008). This temporal variation is maintained by ecological factors such as the phenology of plants and pollinators through the season, pollinators switching plant species and pollinator foraging behavior, yet these factors are being disrupted by global change (Li, Holyoak, and Xiao 2023), leading to phenomena such as phenological mismatch among species (Renner and Zohner 2018). The accelerating pace of global change highlights the importance of studying how ecological networks are changing over time (CaraDonna et al. 2021). Understanding such changes is critical for predicting future dynamics and developing strategies to conserve and manage ecological communities.

**FIGURE 1:**
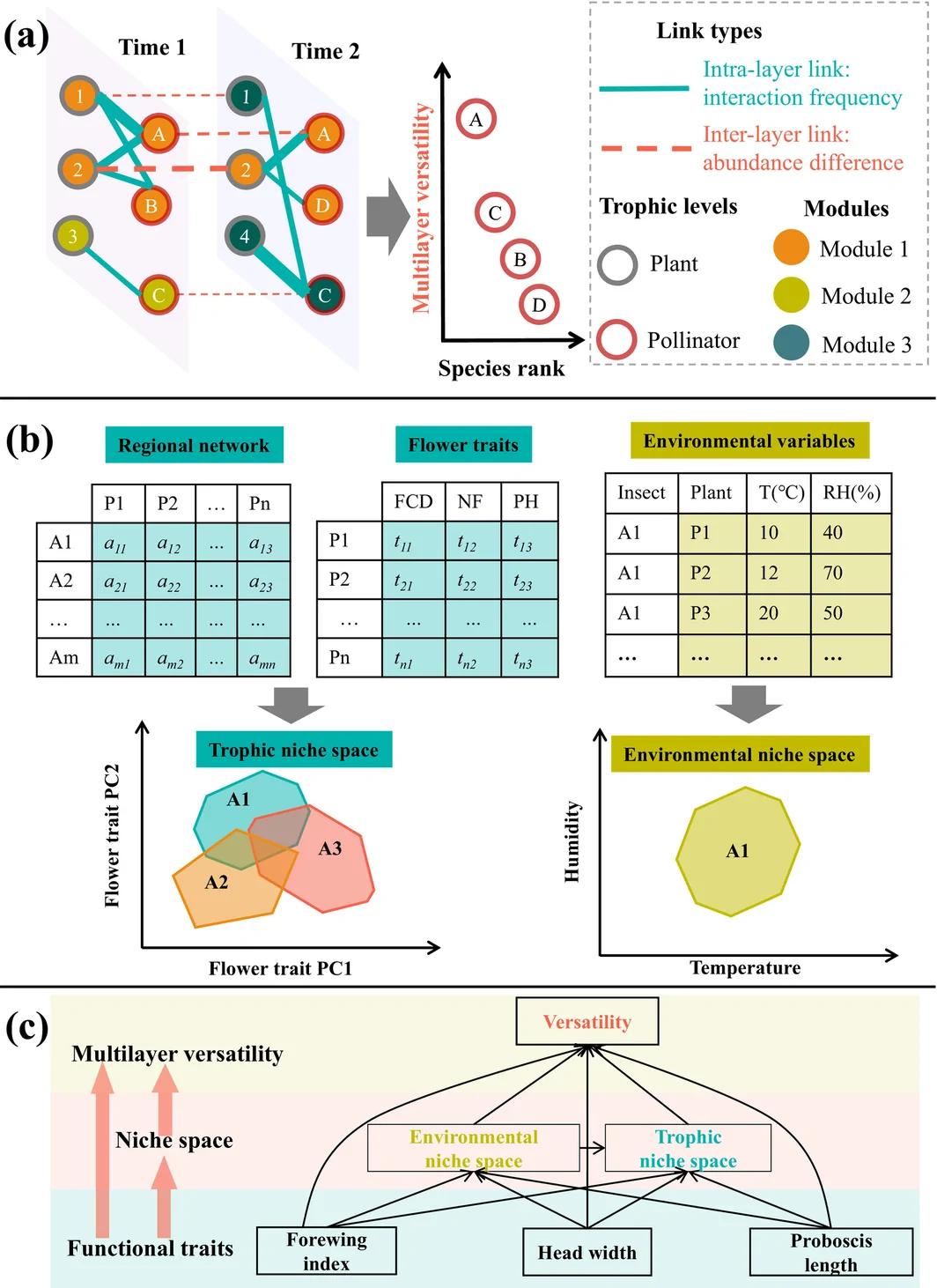
Conceptual framework and hypothesis. (a) Diagram representing the temporal multilayer network for which we built and calculated the modularity and versatility (measured by PageRank centrality). (b) Calculating the trophic and the environmental niche space of a pollinator species based on the “functional diversity” framework in community ecology. FCD, floral corolla depth; NF, number of flower units per plant individual; PH, plant height. (c) Hypothesized path structure for the SEM model showing the effects of functional traits (forewing index, head width, and proboscis length) and trophic and environmental niche space on the pollinators' versatility.

To date, studies on the temporal dynamics of plant–animal networks have typically analyzed multiple disconnected networks, focusing on change in either network structure (Olesen et al. 2008) or quantifying interaction turnover (CaraDonna et al. 2017). While these studies have advanced our understanding of plant–animal community structure, dynamics, and stability, few studies have identified and quantified the role of species that persist across timepoints to connect the network through time. Resasco et al. (2021) synthesized evidence that generalist interactions provide the stable backbone of plant–pollinator networks by persisting across spatial and temporal scales. Studies such as Gruchowski‐Woitowicz et al. (2023) demonstrated which species stabilized network architecture despite seasonal turnover in interactions, and are therefore expected to be most critical for temporal stability in functions (Bramon Mora et al. 2020; Domínguez‐Garcia et al. 2026). Other studies (e.g., Zografou et al. 2020) have concentrated on how phenological mismatches associated with urbanization alter seasonal trajectories of network structure and species roles, or how network properties depend on the temporal scale over which observations are aggregated (Schwarz et al. 2020). Temporal multilayer networks were developed to analyze and overcome such limitations, providing an integrated structure to directly analyze these temporal connections and measure the species' roles in maintaining continuities across time (Hutchinson et al. 2018; Pilosof et al. 2017). To represent a temporal multilayer network, one can define a plant–animal subnetwork (describing intralayer links) at each point or short period of time (a layer). We can then use interlayer links to connect each node (a species) between ordered layers representing connections through time (Costa et al. 2020; Pilosof et al. 2017) (Figure 1a).

Studies on temporal ecological multilayer networks have highlighted two key structural properties: dynamic modularity and species versatility (a.k.a., centrality). While modularity in static networks influences stability by buffering disturbances (Olesen et al. 2007), based on similar logic we would expect that the temporal counterpart of modularity likely governs the persistence of functions and the resilience of the network's structure through time. That is, temporal multilayer modularity reveals a time‐dependent modular structure with modules spanning multiple time layers (Pilosof et al. 2017). Species may interact strongly with different partners at different times, potentially switching among modules over time (flexibility). This causes modules to vary in identity and size (Figure 1a). Species that maintain multiple module affiliations through time are therefore likely vital for sustaining the ecosystem function continuity. Complementing this, species versatility (measured by centrality in networks) identifies species that bridge interactions across multiple time layers, thereby contributing to the network's structural cohesion (De Domenico et al. 2015). These highly versatile species are hypothesized to be crucial for maintaining ecological services, like pollination, through time, and thus may hold high conservation value due to their broad dynamic influence.

**FIGURE 2:**
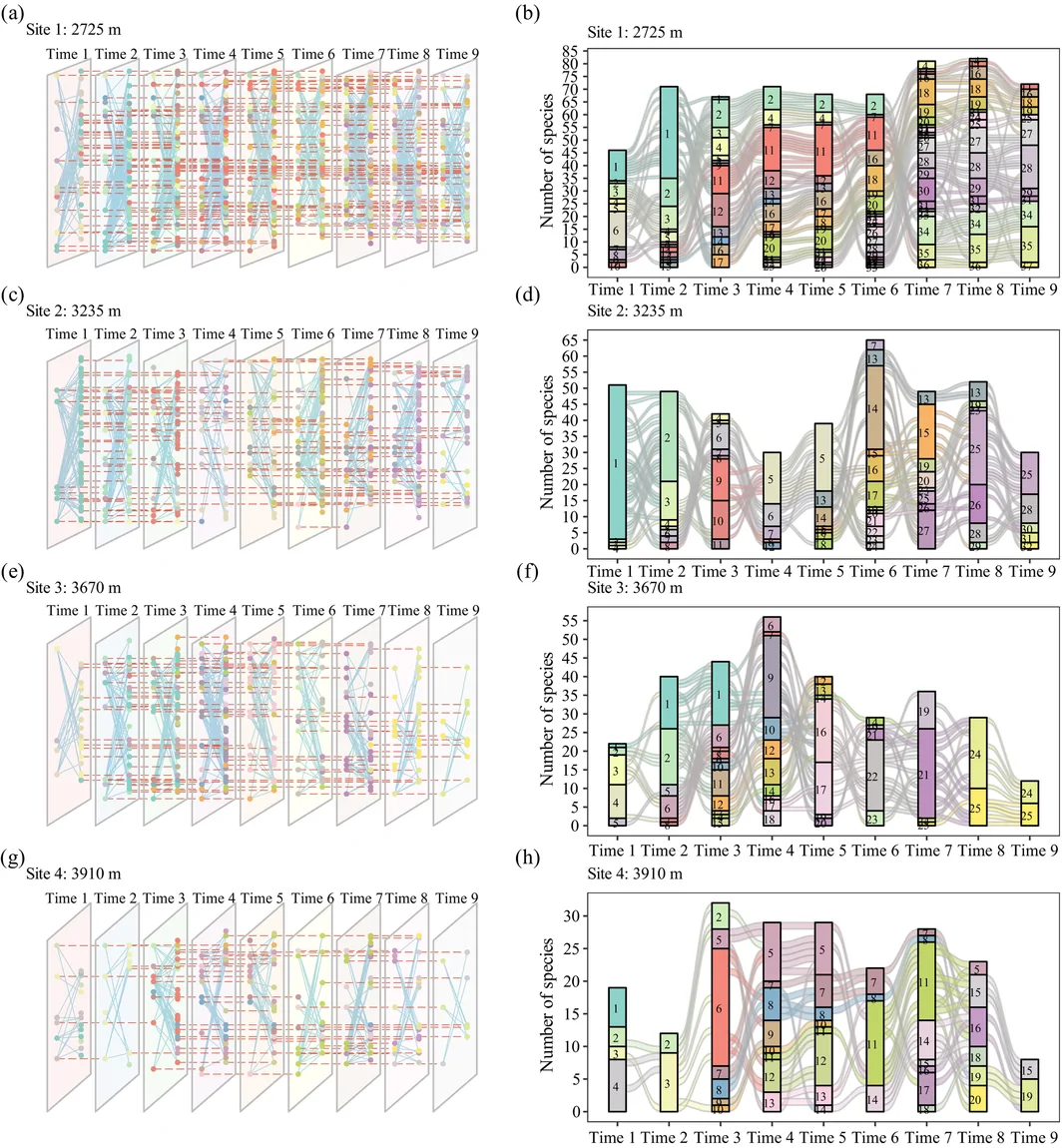
The temporal multilayer pollination networks and alluvial diagrams for the flow of species from different modules among time layers at the four alpine meadows in Yulong Mountain. Temporal multilayer pollination networks at site 1 (a), site 2 (c), site 3 (e), and site 4 (g). Solid lines connecting two nodes indicate intra‐layer links, that is, interactions between plant and pollinator species; and dashed lines indicate interlayer links. The colors of the circle represent different interaction modules. Alluvial diagrams for the flow of species from different modules among time layers of (b) site 1, (d) site 2, (f) site 3, and (h) site 4. Species are clustered in modules, presented in colored blocks. Each line represents a species, and line colors correspond to the module in which the species originates.

Mutualisms involving plants and pollinators are crucial in ecosystem stability and in shaping biodiversity (Bascompte et al. 2006; Chomicki et al. 2019). Plant‐pollinator systems have been shown to be dynamic through seasons in both species and interactions (CaraDonna et al. 2017). Recent studies have also demonstrated that generalist species often persist between temporal snapshots, potentially acting as crucial connectors that link seasonal networks (Resasco et al. 2021; Zografou et al. 2020), and that species with high temporal persistence play key roles in frugivory multilayer networks (Costa et al. 2020). However, studies have not systematically investigated the ecological factors—such as functional traits and niche spaces—that drive species versatility in seasonal multilayer networks. Filling this gap is the central objective of our study. We therefore predict that (1) pollination modules span or vary through the season because species composition changes through the season (Figure 1a), and (2) pollinator versatility is directly related to the breadth of their trophic and environmental niches (Figure 1c).

To test prediction (2), we derive and test three specific hypotheses. First, we hypothesize that pollinator species with a broader trophic niche space will demonstrate greater versatility; trophic niche space is defined as the floral trait space a pollinator can exploit (Figure 1b). In accordance with the concepts of forbidden links and trait matching (Vázquez et al. 2009), we hypothesize that species with broader trophic niches are more likely to exhibit higher versatility over short ecological timescales because they can interact across multiple network modules. Although reciprocal feedbacks are also possible, particularly over evolutionary timescales, our study focuses on this ecological hypothesis.

Second, we hypothesize that a wide environmental niche space (Figure 1b) promotes versatility by sustaining pollinator activity across variable conditions and temporal layers. Animals with broad environmental niche spaces are highly adaptable foragers (Brown 1984; Jiguet et al. 2007; Slatyer et al. 2013), which enables them to persist through time. Consequently, an extended seasonal activity period of a pollinator increases its potential to interact with a wider range of plant species across multiple temporal layers, thus elevating the pollinator's versatility.

Third, we hypothesize that pollinator functional traits relate to their versatility directly or indirectly through the effects of niche spaces. We focus on three key traits that are linked with pollination and foraging behavior: proboscis length (related to trait matching), head width (related to energy requirements), and forewing length (related to the foraging stratum occupied and mobility) (Albrecht et al. 2018). For example, we expect a long proboscis length to enable access to a wider variety of floral tube depths (Stang et al. 2009; Vizentin‐Bugoni et al. 2014), resulting in a broader trophic niche space and higher versatility. Similarly, we predict that greater head width (indicating better body condition) and longer forewings (enhancing mobility) facilitate a broader environmental niche by allowing foraging across a wider range of microclimatic and spatial conditions.

While the network structure in static pollination networks is well documented, our understanding of modularity and the factors of species versatility in temporal multilayer networks remains poor (but see Hervías‐Parejo et al. 2023). In particular, the relationship between pollinator functional traits, niche spaces, and their versatility in a multilayer context has not been empirically tested. Yet, understanding the mechanisms underpinning temporal connectivity is vital for predicting how pollination networks will persist through environmental change. To address this, we applied a temporal multilayer networks approach to a unique dataset of pollen transport networks and associated functional traits and environmental variables during pollinator visits to flowers (i.e., temperature and humidity of the air, which were main abiotic factors that structured flower‐visiting communities at high elevations) collected across nine timepoints (at two‐weeks intervals) through a growing season in four alpine meadows with different elevations (Zhao et al. 2018). The dataset comprises a total of 5369 interactions between 101 plant species and 335 insect pollinator species, allowing us to address whether seasonal dynamics in plant‐pollinator interactions are a general phenomenon across elevations. Our study tests the overarching hypothesis that species with broader niche spaces—facilitated by key morphological traits—are more likely to exhibit higher pollinator versatility, enabling them to connect the network through time.

## 2 Materials and Methods

### 2.1 Study Area

The study was conducted on the eastern slopes of Yulong Mountain, located within the Himalaya‐Hengduan Mountains, SW China. The climate is marked by a rainy season from June through September. The mean annual temperature (from 11 May to 29 September 2012) was 8.9°C–16.6°C and decreases with increased elevation (Zhao et al. 2016). The vegetation includes alpine meadows and forests dominated by *Pinus* species or mixed *Abies*, *Quercus*, and *Rhododendron* species.

### 2.2 Study Design

We collected data at four alpine meadows, located at the Lijiang Forest Ecosystem Research Station along the eastern slopes of the mountain (Zhao et al. 2016). The meadows were: (1) Yushuizhai (site 1), 2725 m a.s.l., 27°00′10″ N, 100°12′05″ E; (2) Haligu (site 2), 3235 m a.s.l., 27°00′09″ N, 100°10′57″ E; (3) Yakou (site 3), 3670 m a.s.l., 27°00′56″ N, 100°10′17″ E; and (4) Diyifeng (site 4, above tree line), 3910 m a.s.l., 27°01′41″ N, 100°11′03″ E. Neighboring meadows were separated by a linear distance of about 2 km.

### 2.3 Plant‐Pollinator Interactions

We studied plant‐pollinator interactions at the four study sites during the 2012 flowering season (early May to early October). We conducted nine censuses at 2‐week intervals for each meadow. For each census, flower‐insect interactions at each site were sampled between 09:00 and 17:00. Sampling was conducted under weather conditions suitable for pollinator activity (e.g., no heavy rain), but we did not set prior quantitative thresholds for temperature or wind speed as well as humidity. Only the visiting insect individuals that made contact with plant reproductive organs or foraged for nectar or pollen were collected (Alarcón 2010). We recorded the date and time for each insect visit. Insect species with no pollen were excluded from all analyses, because including non‐pollinating visitors would construct a visitation network rather than a true pollination network. In our previous study, we showed that this pollen‐based filter captures functional pollination more accurately, although we acknowledge this may exclude some legitimate visitors with undetected pollen (Zhao et al. 2018). We collected specimens of each plant species for identification in the lab. Insects were identified using a combination of both morphology and DNA barcodes (detailed description for insect identification see Zhao et al. (2022)). In total, we conducted 320 h of insect observations during the study period and recorded 5369 pollination events (1396 links) between 101 plant species and 335 pollinator species (a species refers to a distinct combination of morphotype and barcode). A detailed description of sampling was given by Zhao et al. (2016, 2018, 2022).

### 2.4 Species Abundance and Functional Traits

Within each meadow, we established 30 1‐m × 1‐m permanent quadrats to monitor floral abundance and flowering phenology. Once per 2‐weeks, all open flowers and inflorescence/flowering heads (heads for species with small clustered flowers, such as Polygonaceae and Asteraceae) were counted for all species present within each survey (detailed information is given by Zhao et al. (2016)). If a plant species did not occur within the 30 quadrats but we collected pollinators on this plant species, we assigned “1” to its abundance. The abundance of pollinators was used as the sum of the individual visitors sampled from flowers.

We measured three types of traits of plant and pollinator species that are known to structure the mutualistic interactions between flowers and pollinators via trait matching (Albrecht et al. 2018): (1) traits related to size matching and the effectiveness of interactions in achieving pollination, (2) energy traits related to resource provisioning by plants and energy requirements of pollinators, and (3) foraging traits related to foraging stratum and animal mobility. Traits related to size matching were floral corolla depth and proboscis length. Traits related to resource provisioning and energy requirements were the number of flower units per individual plant and pollinator head width. Traits related to foraging stratum and mobility were plant height and the forewing index (i.e., the ratio of forewing length to body length). A detailed description of trait measurement was given by Zhao et al. (2016, 2018, 2022).

### 2.5 Environmental Data

We measured two abiotic environmental variables, temperature and humidity, which are well known to influence the foraging activity and temporal niche partitioning of pollinators (Arroyo et al. 1982; Goodwin et al. 2021; Hoiss et al. 2015; Knop et al. 2018). Each study site was equipped with a HOBO U23‐001 Temperature/Humidity Data Loggers (Onset) that was installed ca. 1.5 m above the ground, out of direct sunlight, and was set to record temperatures and humidity in 10‐min intervals from May to October 2012. We then assigned all flower‐visiting insect individuals foraging temperature and humidity by linking dates and times at a 10‐min resolution.

### 2.6 Trophic Niche Space and Environmental Niche Space

To approximate each pollinator species' fundamental niche, we pooled data from all four meadows through the season, thereby extending the realized niche (observed at a local meadow and time) toward the fundamental niche by sampling varied conditions. Trophic niche space was quantified as the functional richness (FRic) of floral traits, and environmental niche space was defined as the FRic of foraging environmental variables experienced by a pollinator species (Figures 1b and S1). To represent trophic niche space, we used floral corolla depth, the number of flower units per plant, and plant height, while we used temperature and humidity to represent environmental niche space. To calculate trophic niche space, we first computed a Euclidean distance matrix between plant species based on their scaled floral traits, then performed principal component analysis (PCA) to generate plant coordinates along three axes (PC1–PC3). Next, we projected each pollinator species into this floral trait space based on the PCA coordinates of its interacting plant species, calculating the volume of the convex hull encompassing those plant species to represent FRic using the mFD package (Magneville et al. 2021). For environmental niche space, we projected individual pollinator observations into the space defined by temperature and humidity and then calculated the convex hull volume of sampled pollinator individuals to determine FRic using the fundiversity package (Grenié and Gruson 2023). Trophic niche volumes were calculated for the 122 pollinator species that had at least three plant interaction partners, and environmental niche volumes were calculated for a subset of 214 species that had at least three comparable environmental records (temperature and humidity combinations)—both being the minimum requirements for constructing a convex hull. For the remaining species that did not meet the minimum requirements for either niche calculation, we assigned a volume of zero to both trophic and environmental niches. It should be emphasized that this assigned zero does not denote an empty or unoccupied niche, but rather reflects the geometric absence of a measurable volume—effectively representing a highly constrained niche in trait space.

### 2.7 Temporal Multilayer Networks

Site 1 (2725 m) had the most interactions: 2076 (615 links) involving 40 plant and 169 insect species; site 2 (3235 m) had 1335 interactions (372 links) with 30 plants and 123 insects; site 3 (3670 m) had 1311 interactions (289 links) with 33 plants and 93 insects; and site 4 (3910 m) had 647 interactions (165 links) with 27 plants and 58 insects, respectively. Both plant and insect varied over the season (Figures S2–S4). We constructed a temporal pollination multilayer network for each meadow. We used time points as layers and species as nodes. The weight of an intra‐layer link between plant species *i* and pollinator species *j* in layer *t* is the number of visits of pollinator species *j* on plant species *i* (*A*_*ijt*_). The weight of interlayer links of a species was quantified as its abundance in layer “*t* + 1” divided by its abundance in layer *t* (*ω*_*t*,*t*+1_), with the weight set to zero if either abundance was zero. Biologically, the weights of interlayer links reflect temporal dynamics in species phenology and abundances (Costa et al. 2020; Pilosof et al. 2017). We utilized R package “BiMultiNetPlot” (Li 2024) to visualize multilayer networks. Before calculating the multilayer network metrics, we normalized the weights of interlayer‐ and intralayer links by scaling them to range from 0 to 1, separately for each link type. This prevents either link type from dominating metrics due to differences in absolute magnitude (e.g., visitation counts vs. abundance ratios) and follows an approach used for networks with multiple interaction types (Hackett et al. 2019).

### 2.8 Temporal Multilayer Network Modularity

To test the extent to which species belong to multiple, highly interactive modules that can extend within and across layers, we calculated the multilayer modularity and analyzed species module affiliation. We used the R package “infomapecology” (Farage et al. 2021) which used the Infomap method to unveil the module organization of the four temporal multilayer networks. Infomap uses the map equation to measure the minimum number of bits (*L*) that are needed to describe the movement of a random walker in and between the modules of a given network partition *M* modules (Farage et al. 2021). Smaller *L* values indicate higher network modularity. We analyzed the module persistence, and the number of layers persisted for each pollinator species, for each temporal multilayer network. Module persistence was visualized using alluvial diagrams through the R package “infomapecology” (Farage et al. 2021). We tested if the modular structure of the observed multilayer network can be explained by the random process within layer by using the “r2dtable” null model in the “bipartite” package (Dormann et al. 2008). The “r2dtable” null model, utilizing Patefield's algorithm, generates random two‐way tables constrained by observed marginal totals, fixing the number of interactions for each plant and pollinator to preserve heterogeneous interaction distributions and thereby simulating a “neutral” scenario where pollinator species are freely distributed among plant species, resulting in interaction patterns arising solely from these pre‐existing heterogeneous distributions. We ran the null model 100 times to calculate the total number of modules *M*_null_ and the code length *L*_null_. The significance of the observed modularity was compared against the distribution of the modularity of the null networks through *Z*‐scores. We also used an extra intralyer “vaznull” null model and an interlayer null model, given that the marginal total of plant species was positively correlated with observed plant abundance (Figure S7), to test the randomness within layer and among layers, respectively (see detailed information in Supporting Information). At the species level, we calculated the flexibility (number of modules that pollinator species occupy, derived from Bassett et al. (2011)) and species activity (number of layers in which a species was active, defined by Costa et al. (2020)). We tested the association between these two metrics using Spearman rank correlation in every study site to test whether species with higher temporal persistence also exhibited greater flexibility in their network positions. For each multilayer network, we also calculated the species adjustability, which is the proportion of species that change module affiliation between layers (Pilosof et al. 2017). To investigate the temporal dynamics of plant‐insect interactions, we also calculated the temporal beta diversity of interactions and its components between consecutive time points (see detailed information in Supporting Information methods).

### 2.9 Species Versatility

In a multilayer context, Google's PageRank centrality (i.e., versatility) has been used to assess the topological position of a species in the structure of networks (De Domenico et al. 2015). Versatility in multilayer networks quantifies a species ability to participate in diverse interactions and occupy multiple functional roles across different layers. We first formatted the multilayer data into a supra‐adjacency matrix for each temporal multilayer network, then we calculated the multilayer versatility (i.e., Google's PageRank centrality) through the R package “muxViz” (De Domenico et al. 2014). To assess whether observed versatility differed from random expectation, we generated 100 null multilayer networks per site using the r2dtable algorithm and recalculated versatility for each replicate.

### 2.10 Statistics

We used structural equation modeling (SEM) to analyze the direct vs. indirect effects of pollinator functional traits (i.e., head width, forewing index, and proboscis length) and niche spaces (trophic and environmental niche spaces) on multilayer versatility. We tested the potential causal structure of the hypothesized model (Figure 1c) using the R package “lavaan” (Rosseel 2012). Because our study spans only one or a few pollinator generations, we focus on ecological processes operating within species' lifetimes, where pollinators capable of foraging under a wider range of environmental conditions are expected to encounter a greater diversity of flowering plants, thereby increasing opportunities for interactions. All variables included in the SEM were standardized to have a mean of zero and a standard deviation of one prior to model fitting, allowing direct comparison of the relative strength of the estimated path coefficients. Multicollinearity was checked for using Variance Inflation Factors (VIF); all values were < 2, confirming that collinearity did not affect our estimates. Because residual distributions deviated from normality, models were fitted using robust maximum likelihood estimation (MLR), which provides reliable parameter estimates and model diagnostics under non‐normal conditions (Rosseel 2012). We report robust (scaled) model fit statistics, including the Yuan–Bentler scaled *χ* ^2^ test, robust CFI, robust TLI, robust RMSEA, and SRMR.

The forms and magnitudes of the effects of functional traits and niche spaces on multilayer versatility are expected to differ among sites, but such differences can be confounded by site‐to‐site variation in species richness—richer sites inherently have larger interaction pools and greater variance, which can artificially inflate path coefficients via statistical coupling rather than ecological processes. To test this bias, we resampled data 100 times with fixed plant and pollinator species richness (plant = 20 species, pollinator = 50 species) from the original networks for every site and calculated multilayer versatility and reran the same SEM path structure for each resampled iteration. Finally, we tested if the observed path differed from the null models (95% quantiles/CI). Coefficients within these quantiles were then considered robust to richness, confirming an ecological signal not solely driven by richness.

## 3 Results

### 3.1 Multilayer Modularity

Each of the four temporal multilayer pollination networks (Figure 2), each at a different elevation, was modular (Figure S5). Values of the observed map equation statistic *L* differed in observed temporal multilayer pollination networks from the randomly “r2dtable” shuffled networks for every study meadow (*Z*‐score < −2 and *p* < 0.0001 in all cases; Table S1 and Figure S5). Similar results were revealed using the “vaznull” null model (Figure S6) and the interlayer null model (Figure S8). We identified 37 modules in the temporal multilayer network at the low‐elevation site (i.e., site 1), 32 at the mid‐low‐elevation site (i.e., site 2), 25 at the mid‐high‐elevation site (i.e., site 3), and 20 at the high‐elevation site (i.e., site 4) (Table S1 and Figure 2). Module persistence and species seasonal activity through time were short, such that plant‐pollination interactions were dynamically reshaped from May through October (Figure 2). Interaction beta diversity analyses also showed that interaction turnover was consistently high (*β*_WN_: ~0.69–0.75), similar on average among sites, and primarily driven by species turnover (Figure S9). No module spanned all nine temporal layers (May to October) in any of the four alpine meadows (Figures 2 and S10). There were 24.3% (9 modules), of modules in one or two layers in the temporal multilayer networks of site 1. Equivalent figures for sites 2 to 4, respectively, were 68.8% (22), 64.0% (16), and 60.0% (12; Figure S10). The maximum number of layers that modules spanned was eight at site 1, five at site 4, four at site 2 and site 3 (Figure S10).

**FIGURE 3:**
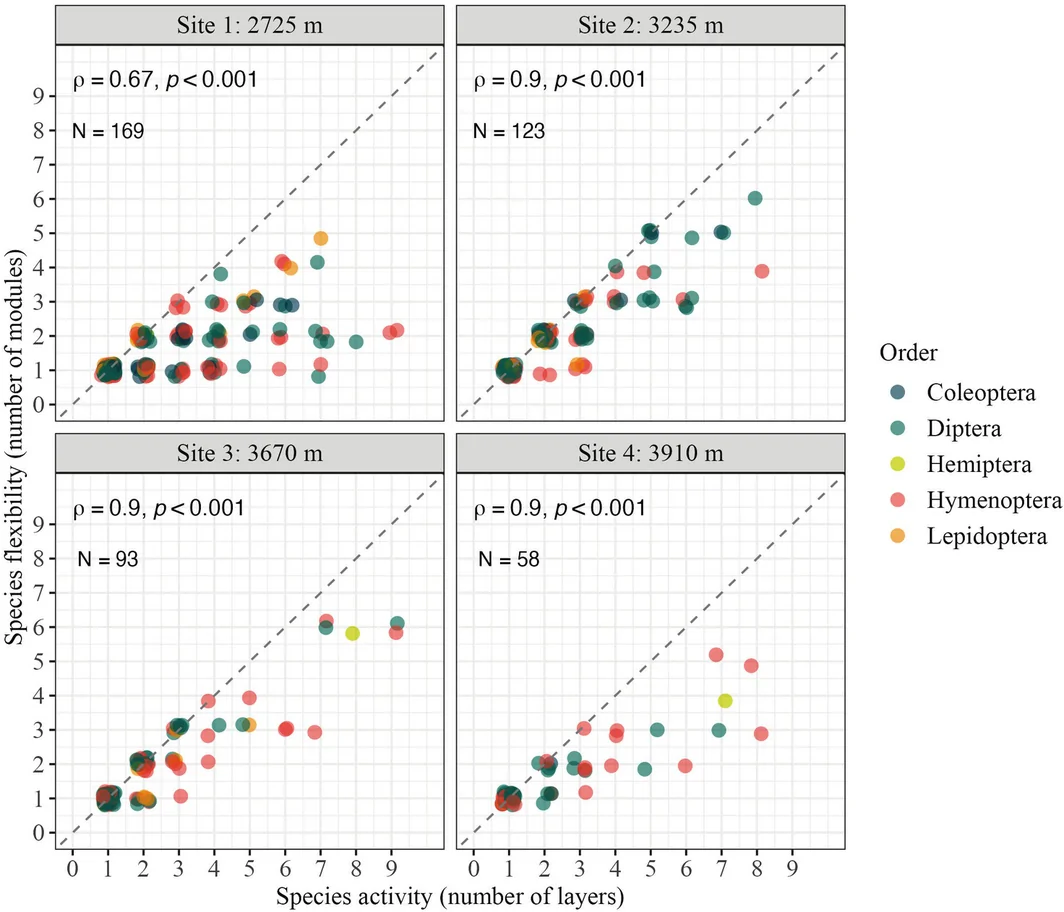
The relationships between pollinator species activity (number of layers persisted) and species flexibility (number of modules occupied). Points are species and jittered. The line within the plot indicates the number of layers persisted equal to the number of modules occupied (i.e., 1:1 line).

On average across sites, the percentage of pollinator species assigned to at least two different modules across layers (i.e., pollinator adjustability) was 47.6% (site 1: 43.2%; site 2: 61.0%; site 3: 48.4%; site 4: 37.9%). The pollinator flexibility (number of modules that pollinator species occupied) and the species seasonal activity (number of layers in which they persisted) were significantly correlated in every study site (site 1: correlation *ρ* = 0.67, *p* < 0.001; site 2‐site 4: *ρ* = 0.90, *p* < 0.001; Figure 3). That is, species with long persistence occupied numerous modules through time. The number of module affiliations of pollinator species ranged from 1 to 6 across the four sites (Figure 3). The pollinator species with the most module affiliations differed among sites except for *Bombus friseanus* , which occupied the most modules at both of the high‐elevation sites (up to six and five in site 3 and 4, respectively).

### 3.2 Pollinator Species' Multilayer Versatility

**FIGURE 4:**
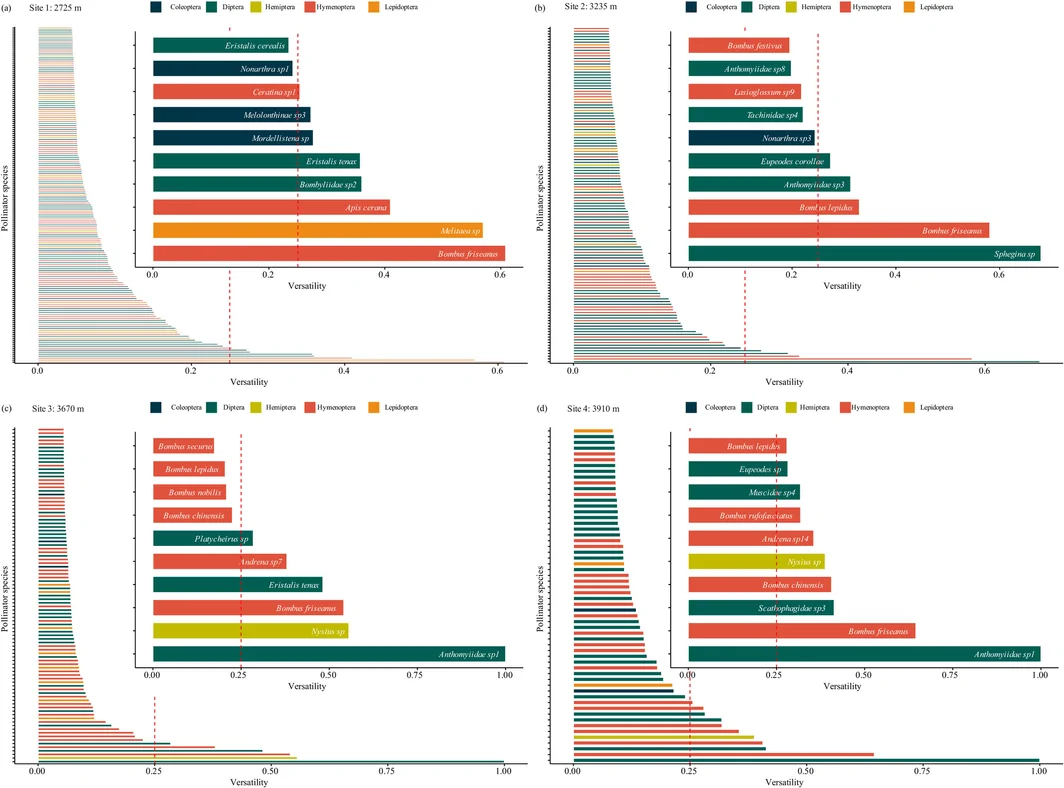
Multilayer versatility of pollinator species. The red dashed line represents the value of versatility equal to 0.25. The top ten most central and versatile pollinator species are indicated with their names. (a) Site 1, (b) site 2, (c) site 3, (d) site 4.

The majority of pollinator species displayed low multilayer versatility (below 0.25), and few pollinators had versatility values greater than 0.25 (4.1%–19.0% of pollinator species [5–11 species] across sites; Figure 4). Across sites, 4–14 species (5.3%–15.1% of pollinator species) had *Z*‐scores ≥ 1.96, indicating that versatility was significantly higher than random expectation. However, these statistically “exceptional” species were not necessarily the same as those with the highest observed versatility values (i.e., versatility > 0.25; Figure S11). The most versatile pollinator species varied among sites (Figure 4), although *Bombus friseanus* was present in all four sites. Pollinator species multilayer versatility was positively correlated with both species flexibility and species seasonal activity (Figures S12 and S13).

### 3.3 The Effect of Functional Traits and Niche Spaces on Pollinator Species' Multilayer Versatility

**FIGURE 5:**
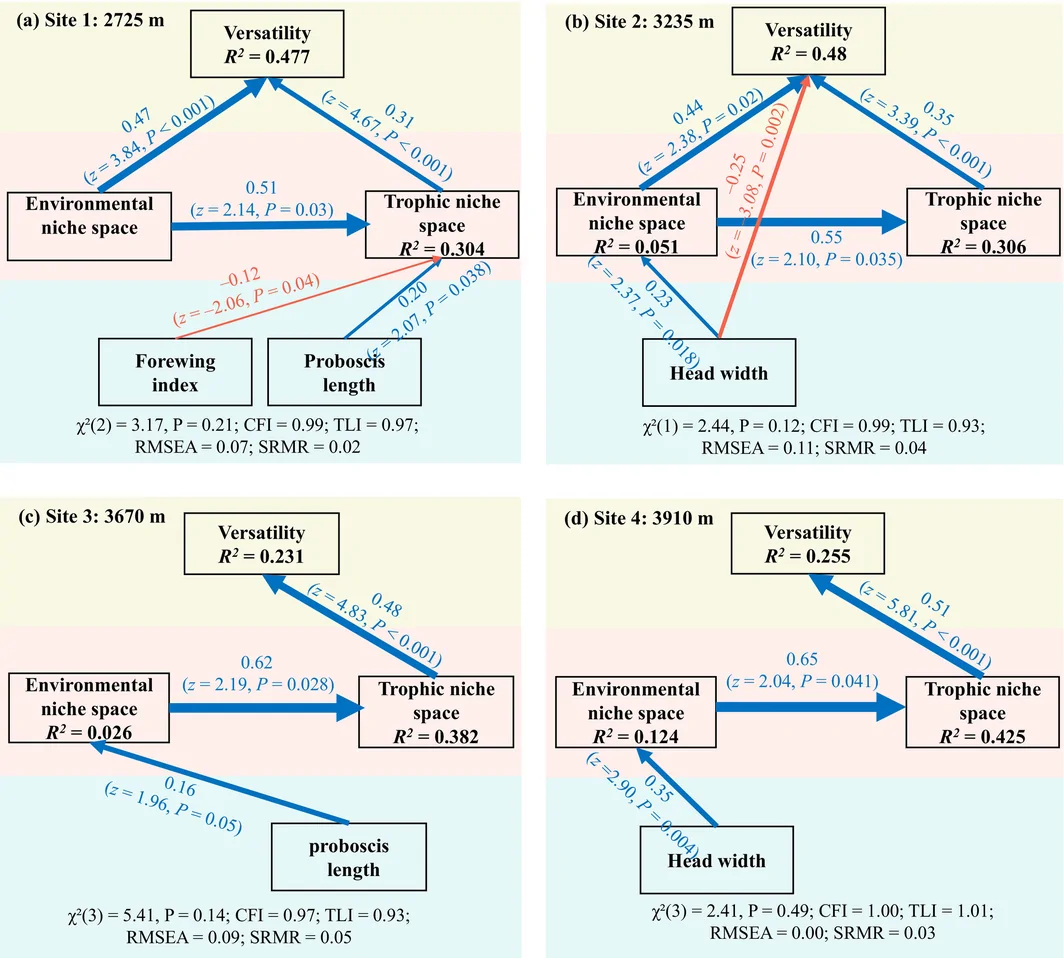
The structural equation models for the direct and indirect effects of functional traits and niche spaces of pollinators on species versatility in each of the four meadows. Only paths that were supported by the variable selection are shown. Blue arrows indicate positive effects, red arrows indicate negative effects. Standardized path coefficients along with arrows were provided. Path widths are proportional to standardized path coefficients.

Across all four sites, structural equation models estimated using robust maximum likelihood (MLR) showed good to excellent fit (Figure 5), and trophic niche space had a significant positive effect on versatility (all *p* < 0.001, Figure 5). Environmental niche space exerted both direct and indirect effects on versatility in sites 1 and 2 but only indirect effects through trophic niche space in sites 3 and 4. The relationships between morphological traits and niche space varied among sites. Proboscis length was positively associated with trophic niche space in site 1 and with environmental niche space in site 3 (Figure 5a,c). Forewing index was negatively associated with trophic niche space in site 1 (Figure 5a). Head width was negatively associated with versatility in site 2, but positively associated with environmental niche space in sites 2 and 4 (Figure 5b,d). The SEMs explained 23%–48% of the variation in versatility and 30%–43% of the variation in trophic niche space, supporting a hierarchical pathway in which functional traits influence environmental and trophic niches, which are subsequently linked to species versatility within pollination multilayer networks. When sample size was standardized across sites, null model results showed that each observed path did not differ compared with the 100 constructed null SEMs for every site, and the differences among sites in SEMs' path coefficients remained similar (Table 1 and Figure S14); hence, in general the results were robust to differences in sample size across sites. An exception was that some path coefficients from the null model overlapped zero in low‐elevation sites (sites 1 and 2), indicating coefficient estimates relied on large sample sizes that were not available at all sites (Table 1 and Figure S14).

**TABLE 1:** Observed standardized path coefficients for the SEM compared with null estimates of standardized path coefficients for the SEM based on 100 random draws with fixed species richness from the original networks.

Site	Path	Observed coefficient	Median of null coefficient	95% CI of null coefficient
Site 1: 2725 m	Environmental niche → Trophic niche	0.515	0.635	[0.273, 0.804]
	Forewing index → Trophic niche	−0.116	−0.107	[−0.317, 0.120]
	Proboscis length → Trophic niche	0.201	0.160	[−0.111, 0.534]
	Environmental niche → Versatility	0.472	0.466	[0.143, 0.766]
	Trophic niche → Versatility	0.314	0.240	[−0.198, 0.607]
Site 2: 3235 m	Head width → Environmental niche	0.225	0.250	[0.028, 0.418]
	Environmental niche → Trophic niche	0.553	0.636	[0.464, 0.78]
	Environmental niche → Versatility	0.440	0.518	[0.195, 0.946]
	Trophic niche → Versatility	0.352	0.275	[−0.358, 0.705]
	Head width → Versatility	−0.250	−0.216	[−0.376, −0.083]
Site 3: 3670 m	Proboscis length → Environmental niche	0.162	0.180	[0.061, 0.328]
	Environmental niche → Trophic niche	0.618	0.665	[0.420, 0.754]
	Trophic niche → Versatility	0.481	0.514	[0.328, 0.693]
Site 4: 3910 m	Head width → Environmental niche	0.351	0.357	[0.255, 0.433]
	Environmental niche → Trophic niche	0.652	0.666	[0.648, 0.713]
	Trophic niche → Versatility	0.505	0.447	[0.288, 0.567]

To evaluate the impact of sampling effort on the relationship between niche spaces and versatility, we performed an additional null model analysis where we standardized the number of individuals for each pollinator species and calculated their niche spaces. These resampled null model paths exhibited directionality similar to what was observed in the empirical data (Figure S15). Most of the observed path coefficients fell within the 95% confidence intervals of the resampled path coefficients distributions. This indicates that our main conclusions are robust to sampling effort, and the directionality of effects is not solely an artifact of the most abundant taxa in the community because they have larger sample sizes, which could have affected their calculated niche spaces.

## 4 Discussion

Previous applications of multilayer networks to pollination have focused on documenting module turnover but have not investigated the relationships between the role of species in maintaining the network structure and their niche spaces. This study directly addressed this question by examining network modularity and assessing how pollinators' trophic and environmental niche spaces relate to their versatility in seasonal pollination networks. Our analysis yielded three key insights into the temporal dynamics of the pollination networks: First, we found that modularity is not a static property but a dynamic one. The composition and size of modules were highly dynamic over time, undergoing continual reconfiguration as interactions turned over. Second, we demonstrated that species persistence is an active, not passive, process. The positive correlation between a species' temporal persistence and its flexibility reveals that long‐lasting species do not simply live in a stable module; they appear to track floral resources through shifts in module affiliation. This dynamic flexibility could be a key behavioral mechanism for persistence in a changing environment, or it could be due to the survival of the pollinators that by chance happen to (randomly) switch modules. Third, and most critically, we identified the functional traits and niche breadths of pollinators that are associated with this key role. We established that a pollinator's versatility—its importance in connecting the network across time—is directly related to the breadth of the pollinator's environmental and trophic niche. This provides a mechanistic link between a species' fundamental niche and its emergent role in maintaining temporal and structural connectivity, a crucial aspect of temporal stability that cannot be assessed with traditional static networks. These patterns were repeated in all four meadows from low to high elevation, providing substantial evidence of the roles of niche spaces in seasonal dynamics of pollination networks. Such results are relevant to the conservation of biodiversity and understanding phenological match/mismatch in a time of global climate change.

Modular structure is a well‐established feature of static mutualistic networks (Li, Tang, et al. 2023; Olesen et al. 2007), and recent studies have begun to investigate how modules persist in temporal multilayer networks (Costa et al. 2020; Hervías‐Parejo et al. 2023; Souza et al. 2022). We confirmed that modules in all four temporal pollination networks were dynamic, continuously reconfiguring across the season. We demonstrated that the well‐documented turnover of both plant and pollinator species and interactions through the season (CaraDonna et al. 2017; Schwarz et al. 2020), which arises from intrinsic phenology and/or environmental variability (CaraDonna et al. 2021), actively and fundamentally reshapes the network's modules through time. We found no module persisted across all layers, with early and late‐season modules becoming entirely distinct (Figure 2). However, our analysis uncovered that temporal changes in modules are not driven solely by species replacement. We found that persistent species maintain their presence by actively tracking floral resources across the season, resulting in dynamic switches in their module affiliations. This “module dynamic” is a response that had been obscured in previous studies of disconnected/static networks. Thus, reconfiguration of modules through the flowering season emerges from two processes: the turnover of species with different phenologies and the interaction rewiring of persistent species (Figure S9). The constant reconfiguration of modules is a structural signature of species successfully foraging in an environment that is changing across the season. Nonetheless, the switching of modules by pollinators of a given species could be behavioral or by chance alone. Empirical evidence supports both the constancy of foraging behavior where generalist pollinators specialize on a given flower species based on handling efficiency (or learning), and that some species optimally switch to other plant species when densities of a preferred species are low (e.g., Chittka et al. 1997; Goulson et al. 1997; Bruninga‐Socolar et al. 2022). Takagi and Ohashi (2025) found that *Bombus ignitus* were more likely to switch to a different plant species if the mixing of offered plant species increased, regardless of the spacing of the preferred plants; in the context of our study this would support pollinators switching modules because of temporal changes in the composition of the plant community. Such potential mechanisms merit field testing.

Network connectivity and centrality metrics are routinely used to identify species with important functional roles (Olesen et al. 2007; Toju et al. 2017). In temporal multilayer networks, species with high versatility (equivalent to centrality in static networks) are expected to be disproportionately important in maintaining continuity of ecosystem functions. Our analysis identified these highly versatile pollinator species, which act as super‐generalists and temporal connectors, within our temporal multilayer pollination networks. The top ten most versatile and important pollinator species were consistently dominated by Diptera and Hymenoptera across all four sites, with Coleoptera species also important in the low‐elevation site. Notably, bumblebees of the genus *Bombus*, such as *B. friseanus* , were key pollinators with high versatility across the season, particularly at high elevations, corroborating their established importance in the Himalaya‐Hengduan Mountains (Eaton et al. 2012; Williams et al. 2009). We also found that a hoverfly (*Sphegina* sp.) and an anthomyiid fly (Anthomyiidae sp1.) were the most versatile species in the high‐elevation sites, aligning with other evidence of flies as important pollinators at high‐elevation sites (Lefebvre et al. 2018). Interestingly, the high versatility and persistence of a hemipteran (*Nysius* sp.) at two sites (site 3 and site 4) suggest that Hemiptera may be underappreciated pollinators in alpine meadow communities. Functionally, these highly versatile super‐generalists, such as *B. friseanus* , connect species both within and between temporal modules and layers. The central role of these species is likely a consequence of their trophic generalism and extended seasonal activity (also see below). This ability to sustain interactions across temporal and ecological contexts would likely predispose species to be able to persist in changing environments. Consequently, we hypothesize that highly versatile pollinators help to maintain network function throughout the flowering season and suggest that they might represent priority candidates for conservation and research. Future studies should test the functional replaceability and vulnerability to environmental change of this species.

Our results strongly support our predictions: pollinator versatility consistently increased with both trophic and environmental niche space across all four meadows. This pattern concords with classical ecological theory positing that broad niche volumes enhance persistence under fluctuating environmental conditions and resource availability (Brown 1984; Gaston and Spicer 2001; Hutchinson 1957). Specifically, we suggest that if pollinator species can use a wide range of flower shapes, they can switch their interactions (plant species) to maintain interactions despite seasonal changes in plant communities and competitor assemblages. Seasonal changes in floral communities and competitor assemblages may shape the observed relationship between environmental and trophic niche space. Notably, this relationship could reflect either correlated ecological responses to contemporary environmental heterogeneity or shared physiological (and evolutionary) adaptations that enable broader environmental tolerance and resource use. While feedback loops between trophic niche breadth and environmental tolerance are theoretically possible, distinguishing such mechanisms would require long‐term or experimental data that are beyond the scope of this study. Our path analyses instead provide evidence for the proximate ecological drivers of versatility. In addition, path analysis revealed that proboscis length indirectly enhanced versatility through its positive effect on trophic niche space (Figure 5), consistent with trait matching and forbidden links as key assembly processes (Sazatornil et al. 2016). The negative relationship between head width and versatility at site 2 may reflect morphological constraints on resource use: smaller‐bodied pollinators are more likely to access floral resources, thereby expanding their interactions across temporal modules. The absence of such a relationship at other sites underscores the context‐dependent nature of trait–network relationships. Beyond this result, the consistency of environmental and trophic niche space relevant relationships across a substantial elevation gradient underscores the robust and general importance of niche spaces and their underlying functional traits in shaping species' role in temporal pollination networks.

While our study reveals a strong influence of traits and niche spaces on species versatility, several limitations merit consideration. First, to avoid using abundance data twice (it was used to weight inter‐layer links), we did not directly test the effects of species abundance on niche spaces and versatility. Instead, we employed a resampling approach to control sample size per pollinator species when calculating niche spaces and then tested the relationship between these spaces and versatility (Figure S15). Although our results suggest that abundance is not the sole driver of versatility, the interpretation of our finding that species versatility is positively correlated with niche spaces should be approached with caution. Specifically, while resampling provides a control, it might not fully capture the complexities of how naturally varying abundance levels affect observed niche breadth. Our sampling method centered on plants, where we systematically collected all observed pollinator insects on plants within our sites. Achieving uniform sampling of pollinator insects in the field is difficult. Second, due to logistical constraints, we primarily focused on species traits and niche spaces and did not incorporate pollinator phylogeny. This may have left unanswered questions regarding potential phylogenetic signals influencing versatility, niches, and traits. Future research could address these limitations by integrating abundance through experimental manipulations (e.g., common garden experiments), alongside continued investigation of species traits and niches, and the explicit inclusion of phylogenetic information. Such a holistic approach would provide a more comprehensive understanding of the drivers of species versatility and the temporal dynamics of pollination networks.

In conclusion, by applying a temporal multilayer network framework, we demonstrated that pollination networks are fundamentally dynamic, with modules continuously reconfiguring across the flowering season. The pollinators most important to this temporal structure were highly versatile species that typically spanned multiple modules, possessed broad trophic and environmental niche spaces, and were often underpinned by key functional traits like proboscis length. Our findings highlight that linking niche spaces and species roles is essential for understanding the persistence and stability of temporal multilayer ecological networks. Future research should extend this multilayer approach to other interaction types (e.g., seed dispersal, herbivory, and predation, etc.) and temporal scales (e.g., hour, day, season, year, and beyond) to understand the multilayer nature of ecological networks in the context of global change.

## Author Contributions

**Hai‐Dong Li:** conceptualization (equal), data curation (equal), formal analysis (lead), funding acquisition (equal), investigation (supporting), methodology (equal), project administration (equal), writing – original draft (lead), writing – review and editing (equal). **Yan‐Hui Zhao:** conceptualization (equal), data curation (equal), formal analysis (supporting), funding acquisition (equal), investigation (lead), methodology (equal), project administration (equal), writing – review and editing (equal). **Marcel Holyoak:** formal analysis (supporting), writing – review and editing (equal). **Zhibin Tao:** investigation (supporting). **Kun Xu:** investigation (supporting). **Zhikun Wu:** investigation (supporting). **Hong Wang:** funding acquisition (equal), writing – review and editing (equal). **De‐Zhu Li:** funding acquisition (equal), writing – review and editing (equal).

## Funding

This work was supported by National Natural Science Foundation of China (32101284 and 32471618), Yunnan Revitalization Talent Support Program “Young Talent” Project (XDYC‐QNRC‐2024‐548), Yunnan Caiyun Postdoctoral Program, Key Basic Research Program of Yunnan Province, China (202101BC070003), Agricultural Experiment Station funding from the USA Hatch Act, Strategic Priority Research Program of the Chinese Academy of Sciences (XDB31000000).

## Conflicts of Interest

The authors declare no conflicts of interest.

## Supporting information


**Table S1:** Values of the observed map equation statistic *L* and the number of modules *M* differed in observed temporal multilayer pollination networks from the randomly shuffled networks.
**Figure S1:** (a) Floral trait space of plant species pool in Yulong Mountain. (b) Using two *Bombus* species to show the pollinator's trophic niche space. (c) Foraging environmental niche space of pollinator species pool in Yulong Mountain. (d) Using two *Bombus* species to show the pollinator's environmental niche space. Niche space was calculated as the functional diversity index (functional richness here was calculated as the volume of the convex hull).
**Figure S2:** Plant flowering phenology of (a) site 1, (b) site 2, (c) site 3, and (d) site 4. R = Time.
**Figure S3:** Pollinator phenology of (a) site 1 and (b) site 2. R = Time.
**Figure S4:** Pollinator phenology of (a) site 3 and (b) site 4. R = Time.
**Figure S5:** Results of the r2dtable null model hypothesis testing of modularity. The *L* value of the map equation (a) and the number of modules (b) of the observed network (dashed vertical lines) are statistically significantly different than those obtained for the null networks in site 1. The *L* value of the map equation (c) and the number of modules (d) of the observed network (dashed vertical lines) are statistically significantly different than those obtained for the null networks in site 2. The *L* value of the map equation (e) and the number of modules (f) of the observed network (dashed vertical lines) are statistically significantly different than those obtained for the null networks in site 3. The *L* value of the map equation (g) and the number of modules (h) of the observed network (dashed vertical lines) are statistically significantly different than those obtained for the null networks in site 4.
**Figure S6:** Results of the “vaznull” null model hypothesis testing of modularity. (a, b) for site 1, (c, d) for site 2, (e, f) for site 3, and (g, h) for site 4. The dashed vertical lines are observed values, and histograms are null values.
**Figure S7:** The correlation between abundance and the marginal total of plants.
**Figure S8:** Results of “shuffle layers” null model hypothesis testing of modularity. (a, b) for site 1, (c, d) for site 2, (e, f) for site 3, and (g, h) for site 4. The dashed vertical lines are observed values, and histograms are null values.
**Figure S9:** Within‐season temporal dynamics of plant‐pollinator interaction turnover (*β*_WN_; purple), the contribution of species turnover to interaction turnover (*β*_ST_; orange), and the contribution of rewiring to interaction turnover (*β*_OS_; green) across the four meadows of the study: (a) site 1, (b) site 2, (c) site 3, (d) site 4.
**Figure S10:** The persistence of each module in time of (a) site 1, (b) site 2, (c) site 3, and (d) site 4. Colors depict module size (i.e., the total number of species in each module). *R* = round.
**Figure S11:** Relationship between observed species versatility and *Z*‑score derived from null model comparisons (100 randomizations per site using the r2dtable algorithm). Each point represents a pollinator species. The horizontal dashed red lines indicate the thresholds for statistical significance (*Z* = ±1.96; *α* = 0.05). Points outside these lines represent species with versatility significantly higher or lower than expected by chance.
**Figure S12:** Positions of pollinator species along multilayer versatility and species activity (number of layers), and the relationship between them.
**Figure S13:** Positions of pollinator species along multilayer versatility and species flexibility (number of modules), and the relationship between them.
**Figure S14:** Observed standardized path coefficients for the SEM compared with null estimates of standardized path coefficients for the SEM based on 100 random draws with fixed network size from the original networks.
**Figure S15:** Observed standardized path coefficients for the SEM compared with null estimates standardized path coefficients for the SEM based on the estimates of the environmental niche and trophic niche via resampling.

## Data Availability

The data and R code for Editor and reviewers were archived in the ScienceDB repository (https://www.scidb.cn/s/QZ7fYv).
