# Supplementary material for: Trophic and Environmental Generalists Maintain Pollinator Network Functional Continuity by Switching Modules Through Time
Source: Ecol Evol. 2026 Sep 17;16(9):e74263. doi: 10.1002/ece3.74263 (PMC13585491; doi:10.1002/ece3.74263)
Supplement: Supplementary file 1 [file ECE3-16-e74263-s001.docx]

**Supplementary Information**

**Trophic and environmental generalists maintain pollinator network functional continuity by switching modules through time**

Hai-Dong Li, Yan-Hui Zhao, Marcel Holyoak, Zhibin Tao, Kun Xu, Zhi-Kun Wu, Hong Wang, De-Zhu Li

**Supplementary Methods**

**S1. Additional null models to test the significance of modularity**

**1) “Vaznull” null model**

We also tested if the modular structure of the observed multilayer network can be explained by random processes by using the ‘vaznull’ null model within layer through the ‘bipartite’ package (Dormann et al., 2008). The ‘vaznull’ null model (Vazquez et al., 2007; Dormann et al., 2009) keeps the connectance equal to the observed network, and builds information from marginal totals into the randomized networks. However, marginal totals are not used as a fixed constraint but only to inform the probability of a certain plant-animal combination when randomly sampling visits. Constrained connectance may represent consequences of specialization. We ran the null model 100 times to calculate the total number of modules M_null_ and the code length L_null_. The significance of the observed modularity was compared against the distribution of the modularity of the null networks through Z-scores.

2) **Interlayer null model:**

We also included a null model that shuffles layers. In this interlayer null model, we assumed that the marginal total is a proxy for the abundance of plant and insect species (it is inappropriate to directly perform shuffling because the interlayer links of plant species and intralayer links come from different datasets that are not comparable). Our solution seems appropriate because the marginal total of plant species was positively correlated with observed plant abundance when putting all nine layers into a Spearman correlation test (Supplementary Fig. 7). This null model randomizing the interactions among layers was used to test whether the observed plant-pollinator interactions were randomly distributed across the nine sampling periods (layers/timepoints). This analysis contained six steps:

1) We began with an observed interaction dataset for each site. This dataset contained four columns: round_id (indicating the sampling time period), plant (plant species), animal (pollinator species), and frequency (with each row representing a single observed interaction, and thus a frequency of 1).

2) We randomized the round_id values within the observed dataset, effectively shuffling the sampling period associated with each observed interaction.

3) For each of the nine sampling time periods (layers), we created a null interaction network (intralayer links). Within each layer, the abundance of each plant and insect species was estimated using the marginal totals (i.e., the sum of interactions for a species). We then used these plant and insect abundances to calculate the interlayer links of plants and animals (i.e., the weight of the connections between species between two consecutive timepoints). Consequently, we yielded a null temporal multilayer network.

4) We performed a modularity analysis on the null temporal multilayer network.

5) This process (steps 2 to 4) was repeated 100 times, generating a distribution of null modularity values (i.e., L values).

6) Finally, we compared the modularity values obtained from the observed networks with the distribution of null values to assess whether the observed modularity pattern deviated significantly from that expected by chance.

**S2. Temporal beta diversity of plant-pollinator networks**

To investigate the temporal dynamics of plant-insect interactions, we calculated the temporal beta diversity of interactions between consecutive rounds (biweek-to-biweek periods) using the framework proposed by Poisot et al. (2012). We calculate the total beta diversity (*β*_WN_) using the Whittaker’s dissimilarity index (Whittaker, 1960):

$$\beta_{WN}=\frac{a+b+c}{(2a+b+c)/2}-1$$

Where ***a*** is the number of links shared by two networks, and ***b*** and ***c*** are the number of unique links in two consecutive rounds respectively. Values for this index range from 0 to 1; higher values indicate higher turnover or greater change between biweekly intervals in who interacts with whom. We further partitioned the total beta diversity into two components using a “common denominator” approach (Fründ, 2023), which is based on Novotny (2009): species turnover-driven component (*β*_ST_) and rewiring (*β*_OS_). The species turnover-driven component, the contribution of species turnover to interaction turnover, is the changes of links (new links and lost links) among species due to species turnover in two consecutive rounds. However, we note that *β*_ST_ is constrained by, but not synonymous with, the turnover of the species themselves, *β*_S_ (see Poisot et al., 2012). Rewiring is the change of links among the shared species in two consecutive rounds-- species were the same but the interactions among them differed in two consecutive rounds. Each of the study meadows yielded multiple values for the contribution of interaction rewiring (*β*_OS_) and species turnover (*β*_ST_) to interaction turnover (one value for each round-to-round transition in each site). We compared the means of each of the two components in each meadow using a two-tailed paired t-test.

**References**

Dormann, C. F., Gruber, B., & Fründ, J. (2008). Introducing the bipartite package: analysing ecological networks. R News, 8(2), 8-11.

Dormann, C. F., Fründ, J., Blüthgen, N., & Gruber, B. (2009). Indices, graphs and null models: Analyzing bipartite ecological networks. The Open Ecology Journal, 2, 7–24.

Fründ, J. (2021). Dissimilarity of species interaction networks: How to partition rewiring and species turnover components. Ecosphere, 12, e03653. <https://doi.org/10.1002/ecs2.3653>

Novotny, V. (2009). Beta diversity of plant–insect food webs in tropical forests: A conceptual framework. Insect Conservation and Diversity, 2, 5–9. <https://doi.org/10.1111/j.1752-4598.2008.00035.x>

Poisot, T., Canard, E., Mouillot, D., Mouquet, N., & Gravel, D. (2012). The dissimilarity of species interaction networks. Ecology Letters, 15, 1353–1361. <https://doi.org/10.1111/ele.12002>

Vázquez, D. P., Melián, C. J., Williams, N. M., Blüthgen, N., Krasnov, B. R., & Poulin, R. (2007). Species abundance and asymmetric interaction strength in ecological networks. Oikos, 116, 1120–1127. https://doi.org/10.1111/j.0030-1299.2007.15828.x

Whittaker, R. H. (1960). Vegetation of the Siskiyou Mountains, Oregon and California. Ecological Monographs, 30, 279–338. https://doi.org/10.2307/1943563

**Supplementary Results**

**S1. Additional null models to test the significance of modularity**

We found that the observed network was more modular than null expectations (Z score of L < –2, Supplementary Fig. 6). However, and interestingly, the number of modules was not significant with vaznull (Supplementary Fig. 6). Results demonstrated plant-animal interactions were not randomly distributed over the course of the season as the observed network was more modular than null expectations (Z score of L < –2, Supplementary Fig. 8).

**S2. Temporal beta diversity of plant-pollinator networks**

Interaction turnover was consistently high (*β*_WN_ in site 1: 0.69; site 2: 0.72; site 3: 0.73; site 4: 0.75), similar on average among sites, and primarily driven by species turnover (Figure S1). The average contribution of species turnover to total interaction turnover exceeded the contribution of rewiring in each meadow/site (site 1: *t*_7_ = -3.50, *P* = 0.01; site 2: *t*_7_ = -5.25, *P* = 0.001; site 3: *t*_7_ = -3.28, *P* = 0.01; site 4: *t*_7_ = -3.42, *P* = 0.01). On average, the species driven component of turnover accounted for 69% of interaction turnover (mean of mean = 69%; mean in site 1: 56%; site 2: 70%; site 3: 73%; site 4: 77%); consequently, the rewiring component averaged 31% and within-meadow averages were between 23% and 44%. The magnitude of interaction turnover in consecutive 2-week periods and its components varied among meadows, and were somewhat idiosyncratic through time in ways that varied among the four meadows (Figure S1).

**Supplementary Table**

**Supplementary Table 1.** Values of the observed map equation statistic *L* and the number of modules *M* differed in observed temporal multilayer pollination networks from the randomly shuffled networks.

| Site | Modularity | Observed value | 95% CI of null models | Z-score | P |
| --- | --- | --- | --- | --- | --- |
| Site 1 | L | 4.83 | (5.72, 5.83) | -35.71 | < 0.0001 |
|  | M | 37 | (14.48, 25) | 5.51 | < 0.0001 |
| Site 2 | L | 3.99 | (4.82, 4.91) | -33.20 | < 0.0001 |
|  | M | 32 | (13, 20) | 7.89 | < 0.0001 |
| Site 3 | L | 3.93 | (4.50, 4.57) | -35.80 | < 0.0001 |
|  | M | 25 | (11, 16) | 8.13 | < 0.0001 |
| Site 4 | L | 3.53 | (4.30, 4.36) | -41.02 | < 0.0001 |
|  | M | 20 | (9, 14.53) | 5.32 | < 0.0001 |

**Supplementary Figures**

**Supplementary Fig 1.** (a) Floral trait space of plant species pool in Yulong Mountain. (b) Using two *Bombus* species to show the pollinator’s trophic niche space. (c) Foraging environmental niche space of pollinator species pool in Yulong Mountain. (d) Using two *Bombus* species to show the pollinator’s environmental niche space. Niche space was calculated as the functional diversity index (functional richness here was calculated as the volume of the convex hull).

**Supplementary Fig 2.** Plant flowering phenology of (a) site 1, (b) site 2, (c) site 3, and (d) site 4. R = Time.

**Supplementary Fig 3.** Pollinator phenology of (a) site 1 and (b) site 2. R = Time.

**Supplementary Fig 4.** Pollinator phenology of (a) site 3 and (b) site 4. R = Time.

**Supplementary Fig 5.** **Results of the r2dtable null model hypothesis testing of modularity.** The L value of the map equation (a) and the number of modules (b) of the observed network (dashed vertical lines) are statistically significantly different than those obtained for the null networks in site 1. The L value of the map equation (c) and the number of modules (d) of the observed network (dashed vertical lines) are statistically significantly different than those obtained for the null networks in site 2. The L value of the map equation (e) and the number of modules (f) of the observed network (dashed vertical lines) are statistically significantly different than those obtained for the null networks in site 3. The L value of the map equation (g) and the number of modules (h) of the observed network (dashed vertical lines) are statistically significantly different than those obtained for the null networks in site 4.

**Supplementary Fig 6. Results of the “vaznull” null model hypothesis testing of modularity.** (a-b) for site 1, (c-d) for site 2, (e-f) for site 3, and (g-h) for site 4. The dashed vertical lines are observed values, and histograms are null values.

**Supplementary Fig 7. The correlation between abundance and the marginal total of plants.**

**Supplementary Fig 8. Results of “shuffle layers” null model hypothesis testing of modularity.** (a-b) for site 1, (c-d) for site 2, (e-f) for site 3, and (g-h) for site 4. The dashed vertical lines are observed values, and histograms are null values.

**Supplementary Fig 9.** Within-season temporal dynamics of plant-pollinator interaction turnover (*β*_WN_; purple), the contribution of species turnover to interaction turnover (*β*_ST_; orange), and the contribution of rewiring to interaction turnover (*β*_OS_; green) across the four meadows of the study: (a) site 1, (b) site 2, (c) site 3, (d) site 4.

**Supplementary Fig 10. The persistence of each module in time of (a) site 1, (b) site 2, (c) site 3, and (d) site 4.** Colors depict module size (i.e., the total number of species in each module). R = round.

**Supplementary Fig 11. Relationship between observed species versatility and Z‑score derived from null model comparisons (100 randomizations per site using the r2dtable algorithm).** Each point represents a pollinator species. The horizontal dashed red lines indicate the thresholds for statistical significance (Z = ±1.96; α = 0.05). Points outside these lines represent species with versatility significantly higher or lower than expected by chance.

**Supplementary Fig 12.** **Positions of pollinator species along multilayer versatility and species activity (number of layers), and the relationship between them.**

**Supplementary Fig 13.** **Positions of pollinator species along multilayer versatility and species flexibility (number of modules), and the relationship between them.**

**Supplementary Fig 14.** **Observed standardized path coefficients for the SEM compared with null estimates of standardized path coefficients for the SEM based on 100 random draws with fixed network size from the original networks.**

**Supplementary Fig 15. Observed standardized path coefficients for the SEM compared with null** **estimates standardized path coefficients for the SEM based on the estimates of the environmental niche and trophic niche via resampling.**
